# Distinct Regulatory DNA Methylation Signatures Across Multiple Sclerosis, Neuromyelitis Optica, and Neurological Post-Acute Sequelae of COVID-19

**DOI:** 10.3390/jcm15134968

**Published:** 2026-06-25

**Authors:** Syed Ilyas Munzir, Daniel B. Hier, Michael D. Carrithers

**Affiliations:** 1 Department of Neurology and Rehabilitation, University of Illinois College of Medicine at Chicago, Chicago, IL 60616, USA; smunz2@uic.edu (S.I.M.); dhier@uic.edu (D.B.H.); 2 Center for Artificial Intelligence and Autonomous Systems, Kummer Institute, Missouri University of Science and Technology, Rolla, MO 65409, USA

**Keywords:** DNA methylation, epigenome-wide association study, multiple sclerosis, neuromyelitis optica, neuro-PASC, long COVID

## Abstract

**Background/Objectives**: Our prior epigenome-wide association study (EWAS) on multiple sclerosis (MS) identified myeloid-associated methylation signatures and an association with enhancer regions. Here we compared differential DNA methylation across three central nervous system inflammatory disorders: MS, neuromyelitis optica (NMO), and neurologic post-acute sequelae of COVID-19 (neuro-PASC). **Methods**: Whole-blood DNA was profiled on Infinium MethylationEPIC arrays. Analyses included EWAS at the CpG level, differentially methylation region (DMR) analysis, and gene regulatory-element enrichment using Locus Overlap Analysis (LOLA). Limma linear models were adjusted for race, EPIC array version, age, sex, disease-modifying treatment class, and blood cell composition. **Results**: All three diseases were associated with broad CpG-level differential methylation. The most robust findings were disease-specific DMR signatures in gene regulatory regions. All three diseases shared Lamin B1-anchored chromatin states as an architectural genomic feature but differed in immune regulatory transcription factor binding sites (TFBS), RNA polymerase (Pol II) occupancy, and DNase accessibility. MS was enriched for TFBS in myeloid CEBPB and SPI1/PU.1 and lymphocyte-associated RUNX3, EBF1, and BATF. MS hypomethylated DMRs were concentrated at active enhancers and myeloid TFBS, suggestive of chronic myeloid activation. NMO showed the clearest promoter and B lymphocyte associated profile. Neuro-PASC was associated with hematopoietic DNase accessibility and TFBS for BATF and EBF1. **Conclusions**: These results suggest that DNA methylation in MS, NMO, and neuro-PASC differ meaningfully in regulatory architecture rather than conforming to a single shared disease-associated methylation model. A long-term goal is to develop immune therapies for newly recognized diseases such as neuro-PASC.

## 1. Introduction

Autoimmune diseases impose a substantial public-health burden. Older studies estimated that selected autoimmune diseases affected approximately 3.2% of the U.S. population; whereas, later reassessment of broader disease groupings and underdiagnosis suggested a prevalence closer to 7.6–9.4% [1,2]. More than 80 autoimmune disorders have been described, and together they account for considerable morbidity, disability, and healthcare utilization. In addition, several autoinflammatory disorders and post-viral conditions that are not classically autoimmune can also produce severe chronic systemic disease.

This study focuses on three inflammatory disorders affecting the central nervous system (CNS): multiple sclerosis (MS), neuromyelitis optica (NMO), and neurologic post-acute sequelae of COVID-19 (neuro-PASC, or neurologic long COVID). MS and NMO are widely regarded as immune-mediated diseases, but they differ substantially in pathobiology [3,4]. By contrast, neuro-PASC is best understood as a post-infectious syndrome associated with persistent immune dysregulation, but there is less definitive evidence that it represents a prototypical autoimmune disorder [5,6,7].

All three conditions can produce substantial and lasting disability. MS remains a leading cause of non-traumatic neurologic disability in young adults [8]. NMO is less common than MS but is often severe and relapses with major visual and spinal cord morbidity [9]. A subset of patients with NMO also demonstrate overlap with systemic autoimmune disease, particularly systemic lupus erythematosus, underscoring the broader immune context in which these syndromes occur [10]. PASC is generally defined by symptoms persisting beyond the acute phase of SARS-CoV-2 infection, commonly beyond 4 weeks [11,12]. Neurologic manifestations of PASC are among the most frequent and disabling and include cognitive dysfunction, headache, fatigue, sleep disturbance, sensory symptoms, and reduced quality of life [13].

Distinct mechanisms are thought to underlie disease initiation in MS and NMO. In MS, evidence from genetic and immunology studies supports a central role for dysregulated peripheral immune responses with additional contribution from CNS-resident myeloid cells, including microglia. Environmental exposures are also important. Longitudinal analysis of more than 10 million U.S. military personnel showed that Epstein–Barr virus infection markedly increased the risk of subsequent MS, strongly implicating EBV as a major upstream determinant of disease in some patients [14]. In contrast, NMO is an autoimmune astrocytopathy in which aquaporin-4 immunoglobulin G (AQP4-IgG) serves as the key serologic biomarker in most affected patients and mediates astrocytic injury with secondary demyelination.

There is also increasing evidence that PASC is associated with persistent immune dysregulation. Prior work has identified altered virus-specific T cell responses and links between immune signatures and neurocognitive symptoms in neuro-PASC cohorts. However, recent studies suggest that innate immune abnormalities may be especially important. PASC has been associated with persistent monocyte and macrophage activation states, including expanded intermediate and CD56+ monocyte subsets, increased non-classical monocytes in patients with cognitive dysfunction, and circulating CD14+ monocyte transcriptional programs linked to systemic inflammation and profibrotic macrophage signatures [15,16]. These findings support a model in which PASC reflects sustained maladaptive immune activation rather than a purely transient post-viral state. Unlike MS and NMO, there are no approved immune therapies for neuro-PASC.

We previously identified novel epigenetic biomarkers in MS in an epigenome-wide association study (EWAS) in a highly diverse population [17]. The strongest associations were with differentially methylated regions (DMRs). These regions contain multiple differentially methylated CpGs unlike single CpG-level associations. Analysis of differential methylation revealed genes linked to hematopoietic proliferation and myeloid-related pathways.

Here, we sought to define both distinct and shared DNA methylation signatures across MS, NMO, and neuro-PASC. Our results suggest that these disorders are best distinguished not simply by isolated methylation differences, but by disease-specific patterns of altered methylation within gene regulatory regions. These findings provide new insights into disease pathogenesis and may support development of biomarker strategies for diagnosis, stratification, clinical monitoring, and development of treatment strategies. Development of new treatments may be particularly relevant to neuro-PASC because no current immune therapies have been established.

## 2. Materials and Methods

### 2.1. Subjects

This was a cross-sectional, case–control study. Seventy subjects with multiple sclerosis (MS), 10 with NMO, 24 with neuro-PASC, and 42 controls with non-inflammatory neurologic disease were enrolled (Table 1). Data from a subset of donors (29 MS and 18 controls) were used in a prior EWAS [17]. All subjects were followed at the University of Illinois Neurosciences Center and were enrolled in the University of Illinois at Chicago (UIC) Neuroimmunology Biobank between August 2018 to May 2024. The UIC Neuroimmunology Biobank was approved by the Institutional Review Board of the University of Illinois College of Medicine (protocol 2017-0520). All subjects provided informed written consent at enrollment.

### 2.2. Inclusion and Exclusion Criteria

**Figure 1:**
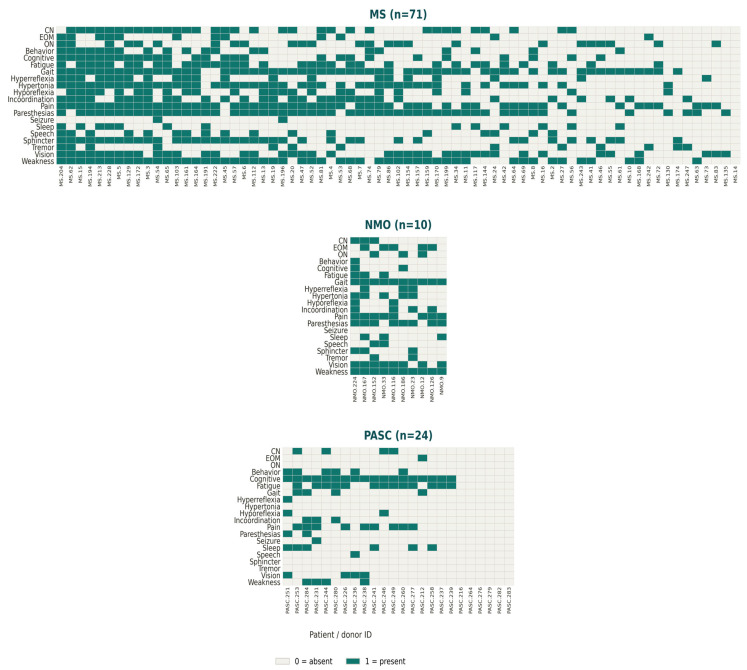
Binary clinical phenotype heatmaps for MS, NMO, and PASC. Separate heatmaps are shown for multiple sclerosis, neuromyelitis optica, and post-acute sequelae of COVID-19. Columns represent individual participants and rows represent binary neurologic signs or symptoms. Cells indicate absence (0) or presence (1) of each phenotype. Within each disease group, participants were ordered by total symptom burden.

Donors (Table 1) were between the ages of 18 and 80 years at the time of enrollment. RECOVER research criteria were used to support classification of PASC/long COVID [18]. None of the patients in the neuro-PASC group were hospitalized for acute COVID-19 infection, and all were referred to our clinic for neurological signs and symptoms (Figure 1). MS and NMO donors met the following criteria: (1) a diagnosis of relapsing-remitting MS based on the 2017 McDonald criteria [19] or a diagnosis of NMO based on the 2015 international consensus diagnostic criteria [20]; (2) no history of relapse within 30 days before sample collection; (3) no history of receiving steroids within 30 days before the sample date; (4) no MRI activity within 30 days before sample collection if MRI was available; and (5) availability of clinical data at the time of sample collection.

The Control group met the following criteria: (1) presentation with a neurologic complaint other than a neuroinflammatory or neurodegenerative disorder; (2) no history of recent ischemic stroke within 6 months before the sample date; (3) no history of systemic autoimmune disease; and (4) ambulatory without assistance at the time of sampling. These individuals enrolled at the same University of Illinois Health neurology clinics and presented with a neurologic complaint. Neuroinflammatory, neurodegenerative, autoimmune, malignant, or infectious etiologies were excluded. Final clinical diagnoses in this group included tension-type headache, migraine, idiopathic peripheral neuropathy, benign positional vertigo, non-specific dizziness, somatic symptom disorder, and functional neurological symptoms. Exclusion criteria for the Control group were active malignancy, recent (<6 months) ischemic stroke, current use of immunomodulating or chemotherapeutic agents, and any history of MS, NMOSD, MOG-antibody disease, other CNS demyelinating disease, autoimmune encephalitis, or post-acute COVID-19 illness.

Exclusion criteria for all subjects were failure to meet the inclusion criteria or use of an immunomodulating or immunosuppressive agent other than disease-modifying treatment for MS or NMO within 6 months before the sample date. All NMO donors met the diagnostic criteria as described above, and 60% (6/10) were anti-aquaporin 4 antibody positive. None of the patients in the study met the diagnostic criteria for MOGAD (Myelin Oligodendrocyte Glycoprotein Antibody-Associated Disease) or were anti-MOG antibody positive.

### 2.3. Clinical Phenotyping

Physician notes completed at the time of enrollment for each patient were gathered from electronic health records. The notes were extracted from REDCap (Research Electronic Data Capture) and converted to JSONL files. Twenty phenotype characteristics were selected based on their frequency and clinical severity in multiple sclerosis cases. All notes were manually annotated.

### 2.4. Whole Blood Methylomics of Genomic DNA

Whole-blood genomic DNA (gDNA) was isolated using the EZ1 Advanced XL automated instrument (Qiagen Cat. No. 9001875; Germantown, MD, USA) with the EZ1&2 DNA Blood 350 μL Kit (Qiagen Cat. No. 951054). Infinium MethylationEPIC BeadChips (Illumina, San Diego, CA, USA) were used to characterize whole-blood genomic DNA methylation in MS, NMO, neuro-PASC, and Control samples. Samples were randomized on the chip. All samples had very high CpG detection rates; therefore, none were removed based on poor detection performance. Raw data in the form of IDAT files were subsequently imported into R (version 4.6.0; R Foundation for Statistical Computing, Vienna, Austria) using the minfi package [21].

### 2.5. Normalization and Filtering

EWAS analysis was performed in R running under macOS Tahoe 26.4.1 (Apple, Cupertino, CA, USA). The workflow was based on code used in our prior study of MS methylomics. Data were filtered and normalized using the preprocessFunnorm function from the minfi and the missMethyl packages [22]. Probes were filtered and removed for low detection values in one or more samples, sex-chromosome-associated probes, CpG sites associated with known single-nucleotide polymorphisms, and probe cross-reactivity [23].

Only probes common to Version 1 and 2 of the bead array assay were retained for analysis. Version 1 (n = 106) and 2 (n = 41) data were filtered and normalized in tandem in minfi and combined into beta value and methylation value (M-value) matrices for downstream analyses. A total of 690,308 CpGs were carried forward for these analyses. As described below, Version was defined as a known covariate in the linear limma model. The effect of this covariate correction is shown in MDS (multi-dimensional scaling) plots in Figure S1.

### 2.6. Linear Model of Differential Methylation

Limma linear models were used as described in the prior study. The differences compared to the prior study were that Treatment (only disease-modifying treatment for MS and NMO), array version (version 1 and 2), and cell composition were included in the linear model. Using the lmFit and eBayes functions from the limma package, models were fit as:

*Design* = *Group* + *Race* + *Version* + *Age* + *Sex* + *Treatment* + *Cell_composition*,

where Design is the M-value indicating the degree of methylation at a given CpG, Group indicates disease condition (MS, NMO, neuro-PASC, or Control), Version is the array version (V1 or V2), and Treatment reflects disease-modifying treatment for MS and NMO only. For the cell composition covariate, estimated blood-cell fractions were first derived from the normalized EPIC matrix using the EpiDISH robust partial correlation (RPC) framework with the cent12CT.m peripheral-blood reference, which estimates 12 immune-cell subsets [24]. Principal component analysis was then applied to the estimated cell-fraction matrix, and the first six principal components (cellPC1-cellPC6) were retained as the dominant cell-composition structure. These variables were incorporated directly into the limma linear model. The full linear model was:

*Design* = *Group* + *Race* + *Version* + *Age* + *Sex* + *Treatment* + *cellPC1* + *cellPC2* + *cellPC3* + *cellPC4* + *cellPC5* + *cellPC6*.

As described in the prior study, removal of unwanted variation (RUV) was used to identify latent variables that should be included in the analyses [25]. The method of Buja and Eyuboglu [26], implemented in the num.sv function of the sva package based on surrogate variable analysis [27], indicated that 14 latent variables should be included in the model. Negative control probes were selected as those with *p* > 0.5 for every effect across the respective disease models. The RUVs function in the RUVSeq package was then used under default parameters to estimate latent unwanted-variation factors [28]. W values were calculated for each latent variable and incorporated into the limma design. The lmfit and ebayes functions in limma were used to calculate the fit.

The contrast matrix for generation of differentially methylated probe (DMP) files was: MS versus Control, NMO versus Control, and neuro-PASC versus Control. All 4 conditions and all three comparator groups were used for the subsequent ANOVA. These ANOVA statistics were included in the DMP files along with delta-beta values and group-specific mean methylation (beta) summaries for individual CpGs. The IlluminaHumanMethylationEPICanno.ilm10b5.hg38 package was used for annotation.

### 2.7. Methylation Heatmap Visualization

For each disease-versus-control contrast, the top 1000 CpGs ranked by FDR-adjusted *p*-value from the RUV-adjusted final-model DMPs were extracted from the β-value matrix (690,308 CpGs × 146 samples). Probes were partitioned into hypermethylated and hypomethylated based on the sign of the case–control mean β difference and stacked with hypermethylated probes on top. β values were row-scaled to per-CpG z-scores and capped at ±2.5 SD to limit the influence of outliers; no column scaling was applied. Samples within each group (case, control) were ordered by hierarchical clustering on Euclidean distance with Ward.D2 linkage; row order was fixed by the hyper/hypo partition rather than clustered. Heatmaps were rendered with pheatmap (v1.0.12) in R 4.5.0 using a diverging RdBu palette from RColorBrewer.

### 2.8. KEGG Pathway Analysis

Gene-level functional enrichment analyses were performed using genes mapped from the EWAS results. The KEGG (Kyoto Encyclopedia of Genes and Genomes) analyses were generated using g:Profiler with g:SCS multiple-testing correction [29]. Over-representation results were obtained for KEGG pathways for each disease contrast, and summary tables were generated from the returned pathway sets.

### 2.9. Analysis of Differentially Methylated Regions (DMRs)

As described in our prior study, the DMRcate package was used to identify DMRs from M-value CpG-level statistics generated by the limma DMP models. Significant DMRs were defined at HMFDR < 0.05. DMR directionality was assigned from the DMRcate meandiff statistic on the β-scale (hypermethylated: meandiff > 0; hypomethylated: meandiff < 0). Gene assignment used the DMRcate overlapping genes field, which lists all RefSeq/UCSC genes intersecting each DMR interval in the hg19 build (IlluminaHumanMethylationEPICanno.ilm10b4.hg19 package).

DMR shortlist construction was performed as follows. For each disease contrast, DMRs were ranked by (i) HMFDR ascending, (ii) |meandiff| descending, and (iii) CpG count descending. The top 50 DMRs by the composite rank were retained, and from these, the top 5 protein-coding-gene DMRs with (a) overlap with at least one LOLACore region set at q < 0.05 and (b) at least one matched-cohort scRNA-seq differentially expressed gene at FDR < 0.05 in any cell type were promoted to the manuscript shortlist.

### 2.10. Gene Regulatory Region Analysis of DMRs Using LOLA

Locus Overlap Analysis (LOLA) of DMRs was performed with the LOLA Bioconductor framework using the hg19 LOLA Core region database to test whether disease-associated DMR intervals were enriched in annotated regulatory elements [30,31]. DMRs were represented as hg19 genomic intervals, enrichment was assessed by Fisher’s exact test with a minimum one-base overlap, and Benjamini–Hochberg correction was applied within each disease-level analysis [32]. The primary universe was defined by the set of regions eligible for DMR discovery in the EPIC-based regional pipeline. For each contrast, LOLA was run separately on the full set of disease-significant DMRs and on directionally stratified subsets (hypermethylated and hypomethylated) using the LOLA Core hg19 collections: ucsc_features, encode_segmentation, encode_tfbs, codex, cistrome_cistrome, cistrome_epigenome, and sheffield_dnase. TFBS, DNase, epigenome, and segmentation-focused collections were reviewed for biological interpretation.

### 2.11. Single-Cell RNA Sequencing (scRNAseq)

Single-cell libraries for RNAseq were constructed from peripheral blood mononuclear cells (PBMC) using the Chromium Next GEM Single-Cell 3′ Kit v3.1 and Chromium Next GEM Chip G (10× Genomics, Pleasanton, CA, USA). Libraries were sequenced on an Illumina NovaSeq 6000. Expression data was analyzed in R software using the Seurat package [33]. The SingleR and celldex packages [34] were used for identification of human peripheral immune cell types using the approach of Monaco et al., 2019 [35]. Using this approach, thirty-one PBMC populations were identified from 9 donors (3 control, 6 MS) that were matched for age, sex, and race. The limma-voom approach in the R-package limma was used to perform differential gene expression analysis within cell types between MS and Control samples [36,37].

### 2.12. Artificial Intelligence Support

Perplexity Computer (Perplexity AI, web version, no public version available; San Francisco, CA, USA) using GPT-5.4 was used as an AI-assisted research platform to support analysis of gene regulatory regions (LOLA), literature review, and assistance with manuscript drafting of gene regulatory (LOLA) methods and results. All generated content was subsequently reviewed and edited by the authors.

## 3. Results

### 3.1. Cohort Characteristics and Binary Clinical Phenotypes Differed Across Disease Groups

The cohort was age and sex-matched and included 70 donors with MS, 10 with NMO, 24 with neuro-PASC, and 42 controls (Table 1). Individuals from the Chicago Metropolitan region were recruited at our Neuroimmunology Clinic at the University of Illinois, Chicago. The population of this region is highly diverse. The ethnicity of the population comprises over 100 distinct ethnic groups. Sixteen percent of the population identifies as Black or African American. In our cohort, 50% (n = 73) of individuals identified as Black or African American.

Clinical phenotyping by manual extraction from the electronic health record was performed. Binary-phenotype heatmaps (Figure 1 and Table S1) showed that MS had the broadest and most heterogeneous clinical pattern. There was substantial inter-individual variability across motor, sensory, visual, and sphincter-related features. By contrast, neuro-PASC demonstrated a lower overall burden of neurologic findings but was relatively enriched for cognitive symptoms. Behavioral abnormalities largely occurred in the subset with cognitive involvement. NMO showed the most clinically restricted pattern, dominated by gait impairment, weakness, pain, paresthesias, and visual features with comparatively limited cognitive-behavioral involvement.

### 3.2. EWAS Identified Broad Disease-Specific CpG-Level Methylation Differences

EWAS was performed on 690,308 CpGs across 146 MS, NMO, neuro-PASC, and Control samples. Analyses were performed in R software as described in our prior MS study. Probes were normalized, filtered, and adjusted for known co-variates including sex, age, race, array version, effects of disease-modifying treatments, and cell composition. MDS plots are shown in Figure S1 to demonstrate the effects of correction of a known covariate (array version). Because of the diversity in ethnicity, comorbidities, non-disease-modifying treatments, and other unknown covariates, corrections were also made for latent variables as described in our prior study.

**Figure 2:**
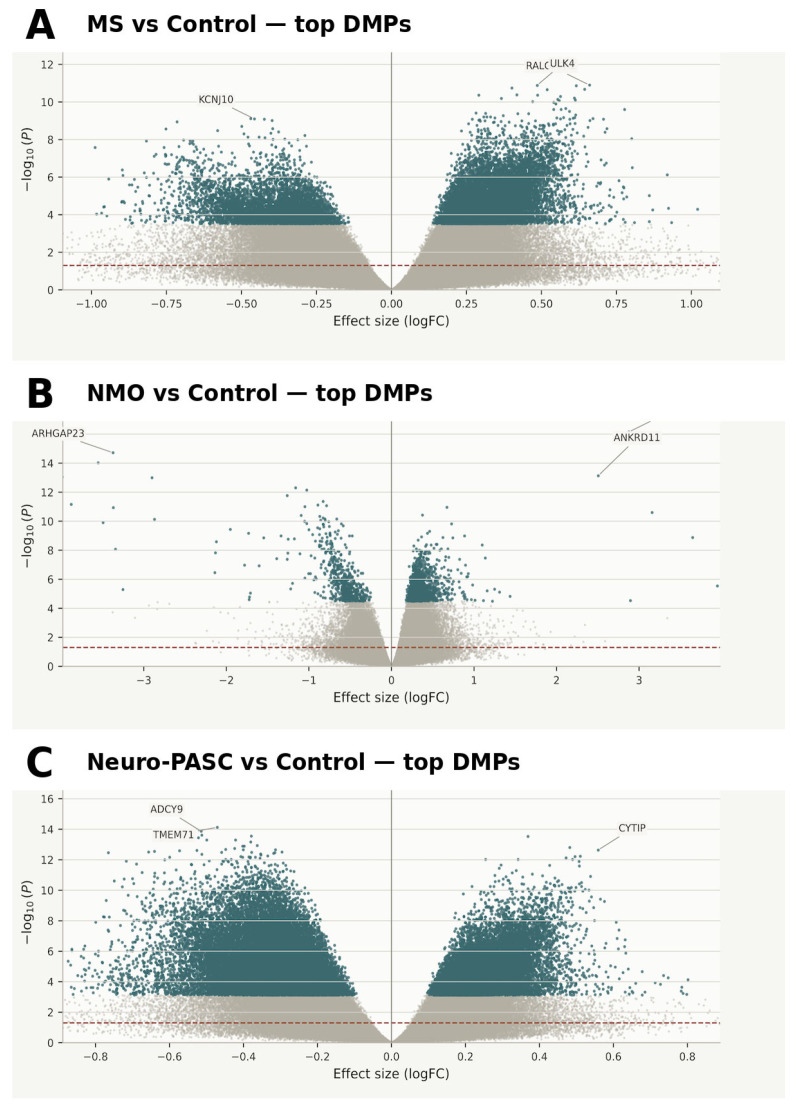
RUV-adjusted final-model EWAS volcano plots for MS, NMO, and neuro-PASC versus controls. Panels (**A**–**C**) show volcano plots of CpG-level log fold-change (logFC) versus −log10 nominal *p* value for MS, NMO, and neuro-PASC versus controls, respectively, for all 690,308 tested CpGs. Teal points denote FDR < 0.01, dashed lines mark nominal *p* = 0.05, and labels identify three top gene-annotated FDR-significant CpGs per comparison. Per-disease methylation heatmaps of the top 1000 FDR-ranked DMPs are provided as Figures S2 (MS), S3 (NMO), and S4 (neuro-PASC) in the Supplementary Materials.

Significantly differentially methylated probes (DMPs) were detected in all three disease-versus-control contrasts at FDR < 0.01. There were 20,681 significant CpGs in MS, 2189 in NMO, and 51,040 in neuro-PASC. These findings indicated the largest CpG-level signal in neuro-PASC, an intermediate signal in MS, and a smaller but still substantial signal in NMO. Volcano plots for each disease-versus-control investigation are shown in Figure 2. Disease-specific methylation heatmaps of the top 1000 DMPs are shown in Figures S2 (MS), S3 (NMO), and S4 (neuro-PASC).

The top-ranked DMPs differed across disease groups. The top 20,000 DMPs for each group are shown in Tables S2–S4. In MS, leading loci mapped to RALGAPA2, ST3GAL5, ULK4, SLC44A2, and SNORD113 with adjusted *p* values as low as 2.50 × 10^−6^. In NMO, the highest-ranking probes included SPATA4, ARHGAP23, PRR22, ANKRD11, ADCY3, DNASE2, SPINK2, and LRRC8B with adjusted *p* values as low as 4.86 × 10^−11^. In neuro-PASC, leading loci included KSR1, RP11-170M17.1, TMEM71, ADCY9, FAM168A, PARVG, MARK2, CCDC69, and KLHL5 with adjusted *p* values as low as 3.98 × 10^−9^.

Cross-disease overlap among FDR-significant CpGs was limited relative to the size of each disease-associated set. Only 144 CpGs were shared across all three disorders (Table S5). One of these common CpGs is associated with FOXP1, a regulator of immune tolerance. Pairwise overlaps were 12,983 between MS and neuro-PASC, 475 between MS and NMO, and 306 between NMO and neuro-PASC. Disease-specific signals remained prominent, including 7369 MS-only CpGs, 1554 NMO-only CpGs, and 37,897 neuro-PASC-only CpGs. Together, these findings indicate that, despite partial overlap, each disorder retained a large disease-specific methylation signature.

### 3.3. Pathway Analyses Provided Supportive Functional Context for the EWAS Results

**Figure 3:**
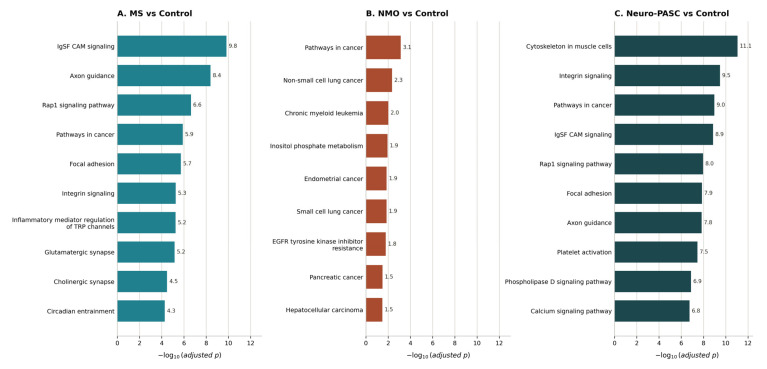
KEGG pathway enrichment across disease comparisons. Horizontal bar plots show the top 10 significant KEGG pathways ranked by adjusted *p* value for each comparison after restricting to pathways meeting the significance threshold used for figure selection. Panels show (**A**) MS vs. Control, (**B**) NMO vs. Control, and (**C**) Neuro-PASC vs. Control. Bar length represents $-{log}_{10}(\mathrm{adjusted}\;p)$, with larger values indicating stronger enrichment.

Kyoto Encyclopedia of Genes and Genomes (KEGG) enrichment analysis based on significant DMPs (FDR < 0.01) identified 46 significant pathways in MS, nine in NMO, and 55 in neuro-PASC (Tables S6 and S7). The most strongly enriched MS pathways included IgSF CAM signaling, Axon guidance, Rap1 signaling pathway, Pathways in cancer, Focal adhesion, and Integrin signaling (Figure 3). In NMO, the significant pathways were Pathways in cancer, Non-small-cell lung cancer, Chronic myeloid leukemia, Inositol phosphate metabolism, Endometrial cancer, Small-cell lung cancer, EGFR tyrosine kinase inhibitor resistance, Pancreatic cancer, and Hepatocellular carcinoma. In neuro-PASC, the leading pathways included Cytoskeleton in muscle cells, Integrin signaling, Pathways in cancer, IgSF CAM signaling, Rap1 signaling pathway, Focal adhesion, Axon guidance, Platelet activation, and Phospholipase D signaling pathway. The greatest degree of overlap occurred between MS and neuro-PASC, whereas NMO had the strongest association with neoplastic pathways.

### 3.4. Region-Level DMR Prioritization Identified Distinct Disease-Specific Regulatory Architectures

Although standard EWAS analysis of DMPs established broad CpG-level disease-associated methylation differences, the main biologically differentiating findings emerged from the analysis of differentially methylated regions (DMRs) and Locus Overlap Analysis (LOLA). These analyses demonstrated distinct regulatory architectures for MS, NMO, and neuro-PASC. Top DMRs for all conditions are shown in Table 2, and all DMRs are listed in Tables S8–S10. Top LOLA hits are shown in Table 3, and all significant LOLA data are in Table S11. A more extensive list of top hypomethylated and hypermethylated LOLA hits is in Tables S12–S17.

### 3.5. MS DMRs Revealed an Immune Regulatory Model in Lymphoid and Myeloid Chromatin Regions

The MS DMR set contained 7570 regions. The overall MS DMR set was approximately balanced between hypermethylation (3731 regions) and hypomethylation (3839 regions). The top DMRs included ARID5B, CTSZ, RAB34, TNFSF12, LTA, SPI1, and the MHC class I region PSMB9/TAP1 (Table 2 and Table S8). Of the top-ranked DMRs, ARID5B, LTA, PSMB9/TAP1, and SEPT9 were hypermethylated, while CTSZ, RAB34, TNFSF12, and the SPI1 promoter region were hypomethylated. All these regions were identified as significant DMRs in our previous study.

LOLA yielded 1702 Benjamini–Hochberg-significant enrichments (q < 0.05) from these DMRs. These enrichments included 529 in the independent hypermethylation-only analysis and 1740 in the independent hypomethylation-only analysis. The LOLA findings for MS demonstrated a two-axis regulatory pattern. Hypermethylated DMRs were strongly enriched in lamina-associated domains (LADs), switch-database transcription start sites (switch DB TSSs), ENCODE database-repressed chromatin segments, and lymphocyte-lineage AP-1 and B cell transcription factor binding sites (BATF, BCL11A, EBF1, IRF4). In contrast, hypomethylated DMRs were strongly enriched in macrophage CEBPB and SPI1 (PU.1) transcription factor binding sites (TFBS), monocyte SPI1 and RUNX1 TFBS, and active enhancer segments across multiple cell lineages (Table 3 and Tables S11–S13). Overall, these results support a model in which MS hypermethylated regions concentrate in heterochromatic/repressed chromatin and lymphocyte AP-1 regulatory patterns, while MS hypomethylated regions concentrate at active enhancers and at myeloid CEBPB and SPI1 binding sites. These results are consistent with chronic monocyte–macrophage priming rather than a broad immunoregulatory signature.

### 3.6. NMO DMRs Supported a Promoter- and Transcription-Complex-Centered Model

The NMO dataset contained 4389 DMRs. The top NMO loci were ARHGAP23, SPATA4, ANKRD11, SLC9A5/FHOD1, BHLHE40, SLC1A5, and SEPT9 (Table 2 and Table S9). The top-ranked NMO candidate was a hypomethylated DMR at ARHGAP23; whereas, the other top DMRs, SPATA4, ANKRD11, BHLHE40, SLC1A5, and SEPT9, were hypermethylated. The overall DMR profile was strongly skewed toward hypermethylation (3396 hypermethylated/993 hypometheylated, ~3.4:1).

The LOLA profile for NMO was the clearest promoter, CpG-island, and transcription-complex-centered pattern among the three diseases (Table 3, Tables S14 and S15). LOLA identified 1665 Benjamini–Hochberg-significant enrichments (q < 0.05) in the combined DMR analysis—1515 in the independent hypermethylation run, and 1392 in the independent hypomethylation analysis. The dominant chromatin findings for hypermethylated DMRs were lamina-associated domains and switchDB TSSs. TFBS included B cell lineage EBF1 and BCOR, RUNX3, and TCF12. The dominant signals for hypomethylated DMRs were RNA polymerase II occupancy (POL2 and POL2A), the histone methylation regulators KDM4A and KDM5B, and FOXA2 and NOTCH1 TFBS. Promoter, CpG-island, and Pol II-associated enrichments substantially outnumbered enhancer-like enrichments. These results supported an NMO regulatory profile concentrated in promoter and CpG-island-rich transcription-associated chromatin with a distinct B cell hypermethylation pattern.

### 3.7. Neuro-PASC DMRs Revealed a Lymphocyte and Myeloid-Chromatin-Linked Model

The neuro-PASC dataset contained 8706 DMRs. The top DMRs comprised CTSZ, RP11-247L20.3/CDKL1, SEPT9, ARID5B, SFRP2, SH2D3C, and RNH1 (Table 2 and Table S10). The overall profile was markedly skewed toward hypomethylation (2545 hypermethylated versus 6161 hypomethylated). Among the top regions, CTSZ, CDKL1, SFRP2, SH2D3C, and RNH1 were hypomethylated, and SEPT9 and ARID5B were hypermethylated.

LOLA identified 2022 Benjamini–Hochberg-significant enrichments (q < 0.05) overall. These included 498 in the hypermethylation-only run and 1988 in the hypomethylation analysis. The leading LOLA regulatory signatures in neuro-PASC were characterized by strong heterochromatic and CpG-dense enrichment. These patterns overlapped with lymphocyte hypermethylation and myeloid hypomethylation patterns. The strongest hypermethylation signals were LaminB1, switchDB TSSs, BATF (shared with MS), EBF1 (shared with NMO), and Sheffield DNase hematopoietic regions. The strongest hypomethylation signals were LaminB1, switchDB TSSs, CpG islands, enhancer segments, and macrophage CEBPB/SPI1 binding sites (Table 3, Tables S16 and S17). Together, these findings support a broad PASC regulatory architecture that combines a lymphocyte AP-1 and B lymphocyte hypermethylation pattern (BATF, EBF1) and a myeloid hypomethylation signature (CEBPB, SPI1) overlaid on strong heterochromatin/CpG-dense regional enrichment.

### 3.8. Common and Distinct Methylation Signatures in MS and Neuro-PASC

Between the three disease conditions, NMO had the most unique methylation signature, while MS and neuro-PASC were most similar. In DMRs, MS and neuro-PASC share a chromatin regulatory pattern in LaminB1 LADs, switchDB TSSs, ENCODE-repressed segments in hypermethylated DMRs, and differential methylation of some enhancer segments. Significant TFBS included macrophage CEBPB and SPI1 overlap in hypomethylated DMRs. They differ in four respects. First, the PASC DMRs are more strongly skewed toward hypomethylation (2545 hypermethylated versus 6161 hypomethylated) than MS (3731 hypermethylated versus 3839 hypomethylated). Second, the hypermethylated DMR TFBS in PASC are more concentrated in lymphoid AP-1 and B lymphocyte patterns with higher odds ratios than MS (BATF OR 10.5 vs. 8.4, EBF1 6.2 vs. 4.0, BCL11A 8.4 vs. 6.0). In addition, there was a markedly stronger hematopoietic DNase accessibility signal (OR ~45 versus ~26) and an additional T lymphocyte TAL1 enrichment. Fourth, the MS hypomethylation DMR signature carries a distinctive multi-stage myeloid SPI1/RUNX1 program (myeloid precursors, monocytes, and macrophages) that is not as prominent in the neuro-PASC top hits. In contrast, neuro-PASC hypomethylated DMRs are more enriched for CpG-island and simple-repeat features. Neuro-PASC retains the MS lymphoid-AP-1 and myeloid-CEBPB/SPI1 axis but in a more hypomethylation-dominant, DNase-accessible, and CpG-island-rich context. Neuro-PASC also has a less strong association with the extensive myeloid-developmental SPI1/RUNX1 pattern that characterizes MS.

### 3.9. Integrated DMR and Single-Cell mRNA Expression Analysis for MS Revealed Correlations for Multiple Genes

A central question in EWAS is how differential methylation correlates with gene expression in specific cell types. To address this question, single-cell RNA sequencing (scRNAseq) analysis of peripheral blood mononuclear cells was performed in a small subset of MS (n = 6) and Control (n = 3) donors. Gene expression in these cell populations was assessed for those associated with the significant DMRs in MS. These associations were examined using the limma-voom method in the R-package limma (top hits in Table 4, full DMR/limma-voom data in Tables S18 and S19; full limma-voom data in Table S20).

A UMAP (Uniform Manifold Approximation and Projection) of the cell populations is shown in Figure S5. Thirty-one cell populations were identified by Seurat analysis. Global integration analysis was performed by intersecting all 7570 significant MS DMRs with all 8811 globally significant limma-voom hits (adjusted global *p* value < 0.05) across the 31 cell types. Of the 8811 significant scRNAseq hits, 1822 (20.7%) involved a gene that was also associated with a significant MS DMR (Table S18). These associations spanned 793 unique genes across 28 of 31 cell types. Of these 1822 (gene and cell type) overlaps, 977 (53.6%) were directionally concordant; 671 hypomethylated DMR demonstrated increased expression, and 306 hypermethylated DMR showed decreased expression (Table S19). There were 692 discordant pairs.

The strongest associations among the top DMRs (Table 2) that are more MS-specific were for two adjacent genes in the MHC class I region that regulate antigen processing and presentation on MHC class I, PSMB9 and TAP1 (MS_DMR_7, chr6:32,819,858–32,821,090, hypermethylated). TAP1 showed significant differential gene expression across six cell types. The strongest associations were an increase in expression in natural killer cells (logFC = +0.23; FDR = 1.45 × 10^−18^) and switched memory B cells (logFC = +0.51; FDR = 1.31 × 10^−2^). PSMB9 was differentially expressed in four cell types. The strongest association was an increase in expression in naive B cells (logFC = +0.33; FDR = 3.23 × 10^−12^). Both genes were up-regulated in MS despite hypermethylation of the shared MS_DMR_7 region, and the two genes were regulated in tandem, as expected for adjacent MHC class I antigen-processing genes. TNFSF12 differential gene expression was more restricted. There were three significant cell types in myeloid populations. The strongest association was an increase in expression in intermediate monocytes (logFC = +0.36; FDR = 2.03 × 10^−4^). ARID5B demonstrated a modest down-regulation limited to naive CD4 T cells (logFC = −0.12; FDR = 3.26 × 10^−2^), concordant with the hypermethylated ARID5B DMR. SPI1, a top hypomethylated MS DMR at chr11:47,399,813–47,401,027, showed paradoxical down-regulation in three myeloid populations (immature macrophages, myeloid dendritic cells, and intermediate monocytes). This finding of a discordant decrease in gene expression suggested a compensatory transcriptional regulation despite enhancer demethylation.

Two genes, IL32 and CTSZ, demonstrated significant DMRs and differential gene expression in MS and were also among the top DMRs for neuro-PASC. The leading common DMR was IL32, which showed differential gene expression in eight cell types in MS. The strongest association was a decrease in expression in natural killer cells (logFC = −0.75; FDR = 3.36 × 10^−56^), concordant with the hypermethylated IL32 DMR (concordant silencing across all five globally significant cell types). CTSZ demonstrated a significant increase in expression in non-classical monocytes (logFC = +0.34; FDR = 1.33 × 10^−4^; plasmablasts: logFC = +0.87; FDR = 3.87 × 10^−1^), concordant with the hypomethylated CTSZ DMR.

Additional associations were observed for significant DMRs that are not on the top DMR list (Tables S18 and S19). A pan-immune de-repression of the S100A8/S100A9 alarmin axis was observed. S100A9 was concordantly hypomethylated and up-regulated in gene expression in 22 of 30 cell types (including NK cells, naive B cells, naive CD4 T cells, Tregs, Th1, Th2, and monocytes, all with adj.P < 1 × 10^−45^) and S100A8 in six. In myeloid populations (monocyte subsets, myeloid dendritic cells, and megakaryocytes), the integration analysis yielded 411 concordant DMR-gene expression pairs, including canonical macrophage/monocyte effector genes that are direct or indirect SPI1/CEBPB targets: CFD (logFC = +0.61; FDR = 2.9 × 10^−81^ in classical monocytes), MNDA, CSF1R, FCGRT, FPR1, OSCAR, NFAM1, IFNGR2, ABCC3, ALDH2, and TSPO. These findings directly link the LOLA macrophage CEBPB/SPI1 hypomethylation enrichment to increased gene expression. Concordant silencing of interferon-induced effectors (IFITM1, IFITM2, B2M), cytotoxic effectors (GNLY, IL32), HLA-F (15 cell types), and the splicing factor SRSF5 was prominent in NK and CD4 T cells (Table 4, Tables S18 and S19).

## 4. Discussion

In our prior study, we identified novel DNA methylation signatures associated with multiple sclerosis in a highly diverse patient population [17]. Here our goals were to compare the MS methylomic signature to two other CNS inflammatory diseases, NMO and neuro-PASC. For MS, integration of the differential methylation with single-cell gene expression analysis was also performed to assess for biologically relevant correlations. One rationale for this study was to identify markers of CNS inflammation that were disease specific rather than a more general signature of neurological autoimmune or autoinflammatory disease. The long-term goals are to distinguish methylation signatures between inflammatory and autoimmune diseases, to develop mechanistic hypotheses of disease pathogenesis, and to identify potential biomarker approaches that could be used in the clinical setting.

One notable finding in the prior study was that there was not a strong association with MHC class II genes as has been observed in other studies of differential DNA methylation in MS [38,39,40]. This finding was replicated here for all three disease conditions. Although it is likely that there are MHC class II associations in CNS inflammatory disease, these findings would be filtered out in our comparison study of MS, NMO, and neuro-PASC because they may be common to all three diseases. In our experimental design, each condition was compared to our Control group, but all three conditions were part of the ANOVA statistical analysis. This approach optimized identification of unique methylation signatures between groups but may have diluted the signals from common regions of differential methylation. Despite the lack of association with MHC class II genes, two genes that regulate antigen processing and presentation on MHC class I, TAPB1 and PSMB9, were identified as having an association with MS in the methylation and gene expression data.

A key strength of the present MS analysis is the substantial concordance of the top region-level findings with our prior MS methylation cohort [17]. The top differentially methylated regions identified here (Table 2) include ARID5B, CTSZ, RAB34, TNFSF12/TNFSF13, and SFRP2, each of which was independently identified among the top 10 DMRs in our prior MS-versus-control comparison. The direction of methylation change is also concordant across cohorts: ARID5B is hypermethylated and CTSZ, RAB34, TNFSF12/TNFSF13, and SFRP2 are hypomethylated in both studies. Because the present analysis includes an EPIC array version covariate, a DMT-class covariate, and an EpiDISH-derived cell-composition adjustment that were not available in the prior study, the recovery of these same DMRs after stricter covariate adjustment constitutes an internal replication of the MS regional methylation signature and increases confidence that the reported MS DMRs reflect disease biology rather than technical or treatment-related variation.

In the prior study, we also identified a gene regulatory pattern in MS that appeared to show enrichment of differentially methylated CpG in enhancer regions rather than promoters. The new region-level analysis here, based on DMR data and LOLA enrichment analysis, indicated that MS, NMO, and neuro-PASC differ in regulatory architecture rather than conforming to a single shared methylation model. Although EWAS demonstrated broad CpG-level differential methylation in all three contrasts, the integrated DMR and LOLA analyses refined those signals into distinct region-level patterns. All three diseases shared a chromatin regulatory signature of a strong LaminB1 association. However, the immune transcription factor and chromatin accessibility patterns differed. MS revealed an immune-regulatory architecture enriched for TFBS in myeloid CEBPB and SPI1/PU.1 and lymphocyte-associated RUNX3, EBF1, and BATF. NMO showed the clearest promoter- and Pol II-centered profile with additional KDM4A, FOXA2, and EBF1/RUNX3/TCF12 enrichments. Neuro-PASC demonstrated the broadest pattern, combining hematopoietic DNase accessibility, EBF1, and BATF onto the shared chromatin regulatory signature.

For MS, these new results no longer support a uniformly enhancer-dominant interpretation. Instead, the stronger signal is one of immune-lineage transcription factor overlap, lamina-associated repressive chromatin, and regulated accessibility at active enhancer regions. The specific transcription factor regulatory region overlaps included hypomethylation at myeloid CEBPB and SPI1 binding sites and hypermethylation at lymphocyte-associated AP1 family of transcription factors. Overall, the results suggest a model of chronic monocyte and macrophage activation.

In contrast, the NMO results were notable for their narrower and coherent methylomic signature. The enrichment of Pol II and promoter-associated features indicates that NMO DMRs are more tightly linked to promoter-proximal transcriptional regulation than MS or neuro-PASC. This interpretation is reinforced by the top LOLA regions in NMO hypomethylated DMRs, which were dominated by Pol II occupancy, the histone demethylase KDM4A, and the promoter-pioneer factor FOXA2. In contrast, the hypermethylated DMR profile combined TFBS for B lymphocyte lineage, EBF1 and BCOR, with LaminB1 lamina-associated repressive chromatin. Taken together, these results suggest that NMO has a relatively narrow promoter and transcription-initiation-centered methylomic signature combined with a prominent B lymphocyte-associated hypermethylation pattern.

Neuro-PASC showed the strongest evidence of broad chromatin-state remodeling. The LOLA profile combined the shared LaminB1 chromatin pattern with hematopoietic DNase accessibility, CpG-island, and enhancer enrichment in hypomethylated DMRs. Immune transcription factor overlap included BATF, EBF1, and CEBPB. BATF overlap was shared with MS hypermethylated DMRs and EBF1 with NMO hypermethylated DMRs, suggesting partial convergence of immune regulation across the three conditions. This combination is compatible with a model in which PASC-associated methylation differences reflect immune-relevant chromatin remodeling rather than a purely promoter-restricted or enhancer-restricted process.

The cross-disease comparison supports distinct regulatory features rather than a single shared methylation program. All three contrasts shared a chromatin-associated pattern of LaminB1 enrichment. However, the immune transcription factor and accessibility signals differed. MS retains the strongest immune transcription factor-rich architecture with lineage-specific CEBPB, SPI1/PU.1, RUNX3, EBF1, and BATF overlap. NMO shows the clearest promoter- and transcription-initiation-centered organization dominated by associations with Pol II, KDM4A, and FOXA2. Neuro-PASC demonstrates the most epigenetically expansive pattern with prominent hematopoietic DNase accessibility and broader immune transcription factor overlap. Although enhancer-linked features remained detectable in all three conditions, promoter, CpG-island, and transcription-associated enrichments were consistently more prominent overall.

A modest number of scRNAseq samples from MS and Control donors were available to assess associations between differential methylation and gene expression in peripheral blood cells. The most prominent concordant signal was de-repression of the alarmin axis S100A8 and S100A9, with S100A9 up-regulated in 22 of 30 cell types adjacent to a hypomethylated DMR. Myeloid-restricted concordant de-repression included CFD, MNDA, CSF1R, FCGRT, FPR1, OSCAR, NFAM1, IFNGR2, ABCC3, ALDH2, and TSPO, providing a transcriptional association with the monocyte–macrophage CEBPB and SPI1/PU.1 LOLA signals.

Against this concordant background, several discordant gene–DMR pairs remained biologically informative. A TAP1 and PSMB9 DMR within a shared hypermethylated region on chromosome 6 showed increased expression across multiple cell types. These findings may be relevant to regulation of antigen processing in the MHC class I pathway for presentation to CD8 T lymphocytes. Although the regulation of these genes usually occurs in tandem, the magnitude of up-regulation was greater for TAP1 than PSMB9. This pattern suggests immune dysregulation in which transcriptional drive overrides local methylation-associated repression that is of unclear significance. A SPI1 DMR showed promoter hypomethylation but decreased expression in three myeloid populations, consistent with compensatory transcriptional regulation in the same lineages where it acts as a master regulator. TNFSF12 and ARID5B showed more lineage-specific alterations in gene expression that may reflect complementary inflammatory regulation and cell differentiation, respectively. Two genes, IL32 and CTSZ, also demonstrated strong DMR signals in neuro-PASC. These bridge candidates between MS and neuro-PASC may be useful for cross-disease comparisons in future studies.

In addition, the gene expression analysis for MS demonstrated possible associations with the LOLA findings of differentially methylated transcription factor binding sites. Prior studies most strongly support a SPI1/PU.1-centered mechanism in myeloid cells [41]. These findings include direct ChIP-based evidence for CTSZ as a SPI1/PU.1 target. There is indirect support for TAP1 and PSMB9 regulation through SPI1-dependent regulation of NLRC5, a known transactivator of MHC class I antigen-processing genes including TAP1 and PSMB9 [42]. TNFSF12 also appears biologically compatible with regulation by the myeloid ETS (E26 transformation-specific) family of transcription factors [43]. Prior work has identified ETS-like GGAA promoter architecture and broader inflammatory chromatin contexts enriched for PU.1 and CEBPB activity. In contrast, to our knowledge, direct evidence linking RUNX3 or TCF3 to regulation of TNFSF12, TAP1, PSMB9, CTSZ, or IL32 has not been identified. The concordant de-repression of myeloid-restricted SPI1/CEBPB target genes (CFD, MNDA, CSF1R, FCGRT, FPR1, OSCAR, NFAM1, S100A8, S100A9) at hypomethylated DMRs provides an independent transcriptional line of support for this mechanistic framework. We speculate that differential DNA methylation of SPI1/PU.1 transcription factor binding sites in myeloid cells provides the strongest mechanistic framework for these gene expression data, and CEBPB may serve as a possible inflammatory co-regulator [44].

Our findings should be interpreted with several constraints in mind. First, the LOLA results should be interpreted as region-level regulatory annotations rather than definitive evidence of direct factor binding at each locus. Second, the prioritized DMR and LOLA hits should be viewed as candidates for downstream validation rather than independent mechanistic confirmation. Third, although disease-modifying therapy (DMT) was modeled as a fixed-effect covariate (eight categorical levels) in all limma linear models so that the MS-versus-control effect estimates reported here are adjusted for DMT class, residual confounding cannot be fully excluded because the untreated MS subgroup is small (n = 9) and exact DMT exposure duration was not uniformly captured. The DMT-class adjustment nonetheless removes the dominant treatment-driven structure in the data. Finally, these results reflect findings in a small cohort at a single institution and need to be validated in larger disease cohorts.

Despite the limitations of this study, the integrated EWAS, DMR, LOLA, and single gene expression framework provides a practical bridge from broad methylome discovery to disease-specific follow-up. For MS, the most informative next experiments may center on immune regulatory networks associated with specific transcription factor binding sites within myeloid cell lineages. For NMO, promoter-oriented methylation–expression analyses are a high-priority next step. For neuro-PASC, integrative follow-up focused on combined lymphocyte and myeloid cell regulation may be especially valuable. More broadly, these data suggest that region-level methylation analysis can reveal biologically meaningful regulatory distinctions that are obscured when interpretation is restricted to CpG-level association alone.

## Figures and Tables

**Table 1:** Cohort characteristics of the analyzed sample set.

Characteristics	MS	NMO	Neuro-PASC	Control
Patients, n	70	10	24	42
Age, years (mean ± SEM)	39.5 ± 1.3	41.0 ± 4.2	44.1 ± 2.5	43.3 ± 2.0
Female, %	75.7	90.0	75.0	76.2
Anti-CD20 treatment, %	22.9	70.0	0.0	0.0
Dimethyl fumarate, %	30.0	0.0	0.0	0.0
Other treatment, %	34.3	10.0	0.0	0.0

Anti-CD20 treatment included ocrelizumab and rituximab. Disease-modifying treatment (DMT) classes among the 70 MS donors were: dimethyl fumarate (n = 21), anti-CD20 monoclonal antibodies (ocrelizumab or rituximab; n = 16), glatiramer acetate (n = 10), untreated (n = 9), interferon-β (n = 5), natalizumab (n = 5), teriflunomide (n = 2), and fingolimod (n = 2). Among the 10 NMO donors: anti-CD20 (n = 7), untreated (n = 2), and glatiramer acetate (n = 1). All 24 neuro-PASC donors and all 42 controls were untreated. DMT class was modeled as an eight-level fixed-effect covariate in all limma linear models.

**Table 2:** Top differentially methylated regions (DMRs) for MS, NMO, and neuro-PASC. DMRs were identified with DMRcate; coordinates are hg19. Direction (Dir.) is hypermethylated (meandiff > 0) or hypomethylated (meandiff < 0). Meandiff is on the β-scale (Δβ). HMFDR refers to the DMRcate HMFDR.

**MS (7570 DMRs at HMFDR < 0.05; 3731 hyper/3839 hypo)**						
Rank	Gene/Locus	Coordinates (hg19)	Dir.	CpGs	HMFDR	Δβ
1	ARID5B	chr10:63,807,168–63,809,170	Hyper	15	4.40 × 10^−26^	+0.048
2	CTSZ	chr20:57,581,903–57,583,709	Hypo	26	1.77 × 10^−18^	−0.019
3	RAB34	chr17:27,044,169–27,045,894	Hypo	21	8.72 × 10^−16^	−0.037
4	TNFSF12/TNFSF13	chr17:7,460,485–7,462,249	Hypo	15	2.89 × 10^−20^	−0.033
5	LTA	chr6:31,539,539–31,543,300	Hyper	25	9.34 × 10^−19^	+0.038
6	SPI1	chr11:47,399,813–47,401,027	Hypo	12	4.19 × 10^−18^	−0.046
7	PSMB9/TAP1	chr6:32,819,858–32,821,090	Hyper	23	7.68 × 10^−22^	+0.018
**NMO (4389 DMRs at HMFDR < 0.05; 3396 hyper/993 hypo)**						
Rank	Gene/Locus	Coordinates (hg19)	Dir.	CpGs	HMFDR	Δβ
1	ARHGAP23	chr17:36,622,717–36,623,419	Hypo	4	1.23 × 10^−22^	−0.094
2	SPATA4	chr4:177,116,576–177,118,196	Hyper	12	6.37 × 10^−24^	+0.029
3	ANKRD11	chr16:89,367,323–89,367,859	Hyper	2	3.50 × 10^−20^	+0.056
4	SLC9A5/FHOD1	chr16:67,280,767–67,282,413	Hypo	12	3.98 × 10^−17^	−0.002
5	BHLHE40/BHLHE40-AS1	chr3:5,018,817–5,020,996	Hyper	15	2.57 × 10^−16^	+0.016
6	SLC1A5	chr19:47,291,295–47,292,253	Hyper	9	2.85 × 10^−12^	+0.012
**Neuro-PASC (8706 DMRs at HMFDR < 0.05; 2545 hyper/6161 hypo)**						
Rank	Gene/Locus	Coordinates (hg19)	Dir.	CpGs	HMFDR	Δβ
1	CTSZ	chr20:57,581,903–57,583,709	Hypo	26	2.19 × 10^−7^	−0.019
2	RP11-247L20.3/CDKL1	chr14:50,863,241–50,865,194	Hypo	14	6.38 × 10^−9^	−0.025
3	SEPT9	chr17:75,445,261–75,447,504	Hyper	12	5.71 × 10^−9^	+0.031
4	ARID5B	chr10:63,807,168–63,809,170	Hyper	15	1.34 × 10^−9^	+0.035
5	SFRP2	chr4:154,710,224–154,712,580	Hypo	32	6.42 × 10^−6^	−0.020
6	SH2D3C	chr9:130,524,573–130,526,319	Hypo	12	3.56 × 10^−8^	−0.045
7	RNH1	chr11:503,270–504,933	Hypo	12	3.11 × 10^−8^	−0.045

**Table 3:** Top chromatin marks and transcription factor binding sites from LOLA (hg19 LOLA Core). Odds ratios and Benjamini–Hochberg-adjusted q-values are shown for the highest-ranked enrichments, separated by DMR direction (hyper- vs. hypomethylated) within each contrast. LOLA q-values are floored at the limit (1.67 × 10^−308^).

**MS Hypermethylated DMRs (3731 DMRs; 529 Enrichments at q < 0.05)**				
**Collection**	**Cell Type/Context**	**Mark/TF**	**Odds Ratio**	**BH-Adjusted q**
ucsc_features	Genome-wide	LaminB1 (LADs)	13.09	<1.67 × 10^−308^
ucsc_features	Genome-wide	switchDB TSSs	106.14	<1.67 × 10^−308^
encode_segmentation	K562	Repressed segments	3.59	6.25 × 10^−289^
encode_segmentation	H1hESC	Repressed segments	3.40	9.70 × 10^−288^
encode_segmentation	HeLa-S3	Repressed segments	3.33	1.81 × 10^−273^
encode_tfbs	GM12878	BATF	8.42	1.01 × 10^−203^
encode_tfbs	GM12878	EBF1	4.01	8.16 × 10^−84^
encode_tfbs	GM12878	IRF4	3.82	7.11 × 10^−65^
**MS Hypomethylated DMRs (3839 DMRs; 1740 Enrichments at q < 0.05)**				
**Collection**	**Cell Type/Context**	**Mark/TF**	**Odds Ratio**	**BH-Adjusted q**
ucsc_features	Genome-wide	switchDB TSSs	488.61	<1.67 × 10^−308^
ucsc_features	Genome-wide	LaminB1 (LADs)	16.22	<1.67 × 10^−308^
encode_segmentation	H1hESC	Enhancer segments	8.04	<1.67 × 10^−308^
encode_segmentation	HUVEC	Enhancer segments	5.75	<1.67 × 10^−308^
codex	Macrophage	CEBPB	12.10	<1.67 × 10^−308^
codex	Macrophage	SPI1/PU.1	10.60	<1.67 × 10^−308^
codex	Monocyte	SPI1	6.31	<1.67 × 10^−308^
codex	Acute myeloid leukemia	SPI1	8.98	<1.67 × 10^−308^
codex	Acute myeloid leukemia	RUNX1	5.25	<1.67 × 10^−308^
**NMO Hypermethylated DMRs (3396 DMRs; 1515 Enrichments at q < 0.05)**				
**Collection**	**Cell Type/Context**	**Mark/TF**	**Odds Ratio**	**BH-Adjusted q**
ucsc_features	Genome-wide	LaminB1 (LADs)	11.65	<1.67 × 10^−308^
ucsc_features	Genome-wide	switchDB TSSs	375.80	<1.67 × 10^−308^
encode_tfbs	GM12878	EBF1	4.89	3.19 × 10^−132^
encode_tfbs	GM12878	RUNX3	3.15	1.30 × 10^−111^
codex	B cells (germinal center)	BCOR	2.96	6.37 × 10^−111^
encode_tfbs	GM12878	TCF12	4.45	1.48 × 10^−107^
encode_tfbs	GM12878	PAX5	3.73	3.87 × 10^−90^
codex	B cells	BCL6	3.23	1.50 × 10^−100^
**NMO Hypomethylated DMRs (993 DMRs; 1392 Enrichments at q < 0.05)**				
**Collection**	**Cell Type/Context**	**Mark/TF**	**Odds Ratio**	**BH-Adjusted q**
ucsc_features	Genome-wide	switchDB TSSs	738.51	<1.67 × 10^−308^
ucsc_features	Genome-wide	LaminB1 (LADs)	12.90	<1.67 × 10^−308^
codex	Embryonic stem cell	KDM4A	17.43	<1.67 × 10^−308^
codex	Endoderm	FOXA2	17.28	<1.67 × 10^−308^
encode_tfbs	HUVEC	Pol2	19.46	<1.67 × 10^−308^
encode_tfbs	Glioblastoma	Pol2	17.89	<1.67 × 10^−308^
encode_tfbs	MCF-7	Pol2	15.86	<1.67 × 10^−308^
**Neuro-PASC Hypermethylated DMRs (2545 DMRs; 498 Enrichments at q < 0.05)**				
**Collection**	**Cell Type/Context**	**Mark/TF**	**Odds Ratio**	**BH-Adjusted q**
ucsc_features	Genome-wide	LaminB1 (LADs)	10.98	<1.67 × 10^−308^
ucsc_features	Genome-wide	switchDB TSSs	78.26	<1.67 × 10^−308^
encode_tfbs	GM12878	BATF	10.46	5.49 × 10^−194^
sheffield_dnase	Hematopoietic	DNase hypersensitivity	44.82	6.03 × 10^−147^
encode_segmentation	HeLa-S3	Repressed segments	2.75	5.52 × 10^−135^
encode_tfbs	GM12878	EBF1	6.25	1.95 × 10^−130^
encode_tfbs	GM12878	BCL11A	8.40	4.60 × 10^−114^
encode_tfbs	GM12878	IRF4	4.94	2.95 × 10^−74^
**Neuro-PASC Hypomethylated DMRs (6161 DMRs; 1988 Enrichments at q < 0.05)**				
**Collection**	**Cell Type/Context**	**Mark/TF**	**Odds Ratio**	**BH-Adjusted q**
ucsc_features	Genome-wide	LaminB1 (LADs)	15.62	<1.67 × 10^−308^
ucsc_features	Genome-wide	switchDB TSSs	488.46	<1.67 × 10^−308^
ucsc_features	Genome-wide	CpG islands	3.05	<1.67 × 10^−308^
encode_segmentation	H1hESC	Enhancer segments	7.86	<1.67 × 10^−308^
codex	Macrophage	CEBPB	11.57	<1.67 × 10^−308^
codex	Macrophage	SPI1/PU.1	9.15	<1.67 × 10^−308^
codex	Acute myeloid leukemia	RUNX1	4.64	<1.67 × 10^−308^

**Table 4:** Top genes from single-cell mRNA sequencing that intersect with top DMRs from MS. The strongest cell type and its logFC and FDR are taken from the limma-voom global FDR (adj.P.Val.gbl_DiseaseMS) and refer to the most-significant cell type for that gene. Concordance refers to the direction of the DMR (hyper vs. hypo) versus the direction of the expression change in the most-significant cell type. # Sig cell types counts cell types passing global FDR < 0.05.

Gene	MS DMR	Region (hg19)	DMR Dir.	# Sig Cell Types	Strongest Cell Type	logFC	FDR	Concordance
IL32	DMR_188	chr16:3,114,847– 3,115,809	Hyper	8	Natural killer cells	−0.75	3.36 × 10^−56^	Concordant silencing
TAP1	DMR_7	chr6:32,819,858–32,821,090	Hyper	6	Natural killer cells	+0.23	1.45 × 10^−18^	Discordant (hyper + up)
PSMB9	DMR_7	chr6:32,819,858–32,821,090	Hyper	4	Naive B cells	+0.33	3.23 × 10^−12^	Discordant (hyper + up)
SPI1	DMR_6	chr11:47,399,813–47,401,027	Hypo	3	Immature macrophages	−0.36	7.34 × 10^−5^	Discordant (hypo + down)
TNFSF12	DMR_4	chr17:7,460,485– 7,462,249	Hypo	3	Intermediate monocytes	+0.36	2.03 × 10^−4^	Mixed-DMR (both directions)
CTSZ	DMR_2	chr20:57,581,903–57,583,709	Hypo	1	Non-classical monocytes	+0.34	1.33 × 10^−4^	Concordant de-repression
RAB34	DMR_3	chr17:27,044,169–27,045,894	Hypo	2	Intermediate monocytes	+0.32	1.23 × 10^−3^	Concordant de-repression
ARID5B	DMR_1	chr10:63,807,168–63,809,170	Hyper	1	Naive CD4 T cells	−0.12	3.26 × 10^−2^	Concordant silencing

Note. TAP1 and PSMB9 share the same MS_DMR_7 region at chr6:32,819,858–32,821,090 (hypermethylated). Both genes are up-regulated in MS across multiple cell types, consistent with their coordinated regulation as adjacent MHC class I antigen-processing genes; both pairs are formally classified as discordant_hyper_up under the methylation–expression concordance framework. SPI1 demonstrates strong hypomethylation at its own promoter region but shows down-regulation in three myeloid populations (discordant_hypo_down), suggesting compensatory transcriptional regulation. The pan-immune S100A8/S100A9 alarmin axis is shown in Table S19.

## Data Availability

The original contributions presented in this study are included in the article/Supplementary Materials. Further inquiries can be directed to the corresponding author.

## Supplementary Materials

The following supporting information can be downloaded at: https://www.mdpi.com/article/10.3390/jcm15134968/s1, Figure S1. MDS plots of covariate correction for array Version number; Figure S2. Heatmap of the top 1000 MS-versus-control differentially methylated probes (DMPs); Figure S3. Heatmap of the top 1000 NMO-versus-control differentially methylated probes (DMPs); Figure S4. Heatmap of the top 1000 neuro-PASC-versus-control differentially methylated probes (DMPs); Figure S5. Single cell RNAseq analysis of peripheral blood mononuclear cells (PBMC); Supplementary Table S1. Binary phenotype prevalence by symptom; Supplementary Table S2. Top 20,000 DMPS for MS; Supplementary Table S3. Top 20,000 DMPs for NMO; Supplementary Table S4. Top 20,000 DMPs for neuro-PASC; Supplementary Table S5. Interersect all 3 groups. 144 DMPs; Supplementary Table S6. KEGG summary; Supplementary Table S7. Top KEGG pathways for each condition. Supplementary Table S8. MS DMRs; Supplementary Table S9. NMO DMRs; Supplementary Table S10. neuro-PASC DMRs; Supplementary Table S11. LOLA all results for all conditions; Supplementary Table S12. Top Hypermethylated LOLA for MS; Supplementary Table S13. Top Hypomethylated LOLA for MS; Supplementary Table S14. Hypermethylated LOLA for NMO; Supplementary Table S15. Hypomethylated LOLA for NMO; Supplementary Table S16. Hypermethlyated LOLA for neuro-PASC; Supplementary Table S17. Hypomethylated for neuro-PASC; Supplementary Table S18. All MS limma-voom overlaps with DMRs; Supplementary Table S19. Concordant limma-voom overlaps with MS DMR; Supplementary Table S20. All limma-voom results.

## Abbreviations

The following abbreviations are used in this manuscript:

AI	artificial intelligence
AQP4-IgG	aquaporin-4 immunoglobulin G
BIOS	Biobank-based Integrative Omics Study
CGI	CpG island
CNS	central nervous system
CpG	cytosine-phosphate-guanine dinucleotide
DMPs	differentially methylated positions
DMRs	differentially methylated regions
DNA	deoxyribonucleic acid
DNase	deoxyribonuclease hypersensitivity annotation
EBV	Epstein–Barr virus
ENCODE	Encyclopedia of DNA Elements
EPIC	Illumina Infinium MethylationEPIC array platform
EpiDISH	Epigenetic Dissection of Intra-Sample-Heterogeneity
eQTM	expression quantitative trait methylation
EWAS	epigenome-wide association study
FDR	false discovery rate
gDNA	genomic DNA
GO	Gene Ontology
GPT-5	Generative Pre-trained Transformer 5
KEGG	Kyoto Encyclopedia of Genes and Genomes
LOLA	Locus Overlap Analysis
MAPK	mitogen-activated protein kinase
MRI	magnetic resonance imaging
MS	multiple sclerosis
NIH	National Institutes of Health
NMO	neuromyelitis optica
PASC	post-acute sequelae of COVID-19
PI3K-Akt	phosphoinositide 3-kinase and protein kinase B signaling pathway
QTL	quantitative trait locus
RECOVER	Researching COVID to Enhance Recovery
REDCap	Research Electronic Data Capture
RNA	ribonucleic acid
RUV	removal of unwanted variation
RUVSeq	Removal of Unwanted Variation from RNA-Seq data framework
SARS-CoV-2	severe acute respiratory syndrome coronavirus 2
SEM	standard error of the mean
TF	transcription factor
TFBS	transcription factor binding site
TSS	transcription start site
UIC	University of Illinois Chicago
UCSC	University of California, Santa Cruz
