# Supplementary material for: Distinct Regulatory DNA Methylation Signatures Across Multiple Sclerosis, Neuromyelitis Optica, and Neurological Post-Acute Sequelae of COVID-19
Source: J Clin Med. 2026 Jun 25;15(13):4968. doi: 10.3390/jcm15134968 (PMC13362688; doi:10.3390/jcm15134968)
Supplement: Supplementary file 1 [file jcm-15-04968-s001.zip › jcm-4336141-supplementary material/Supplemental Figures/Figure S1.pdf]

**A**

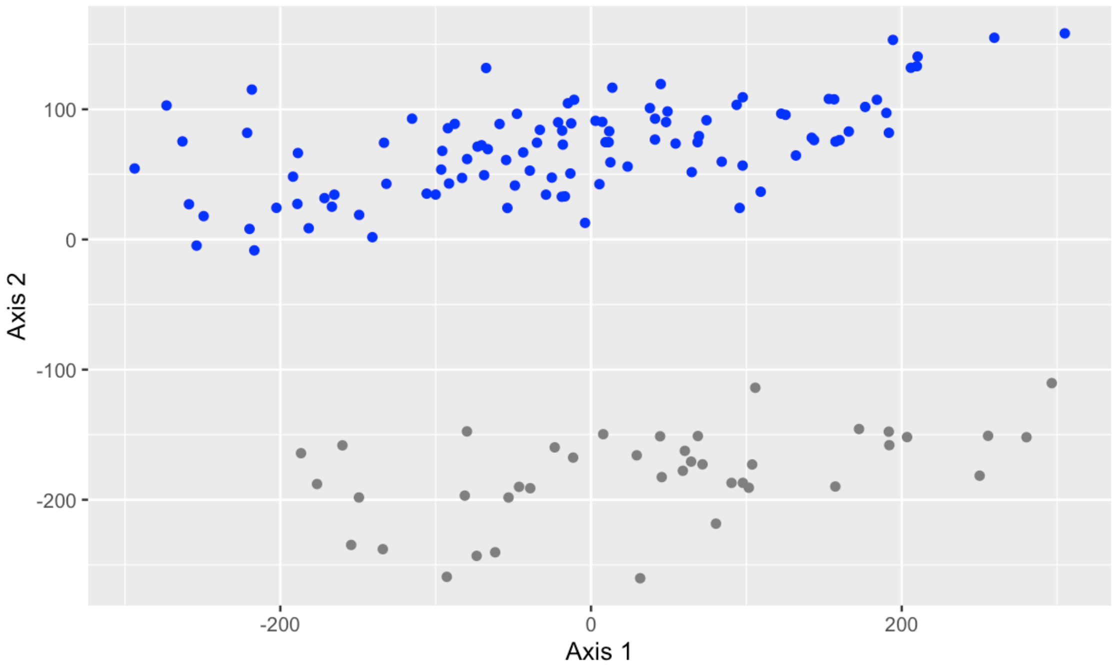


**B**

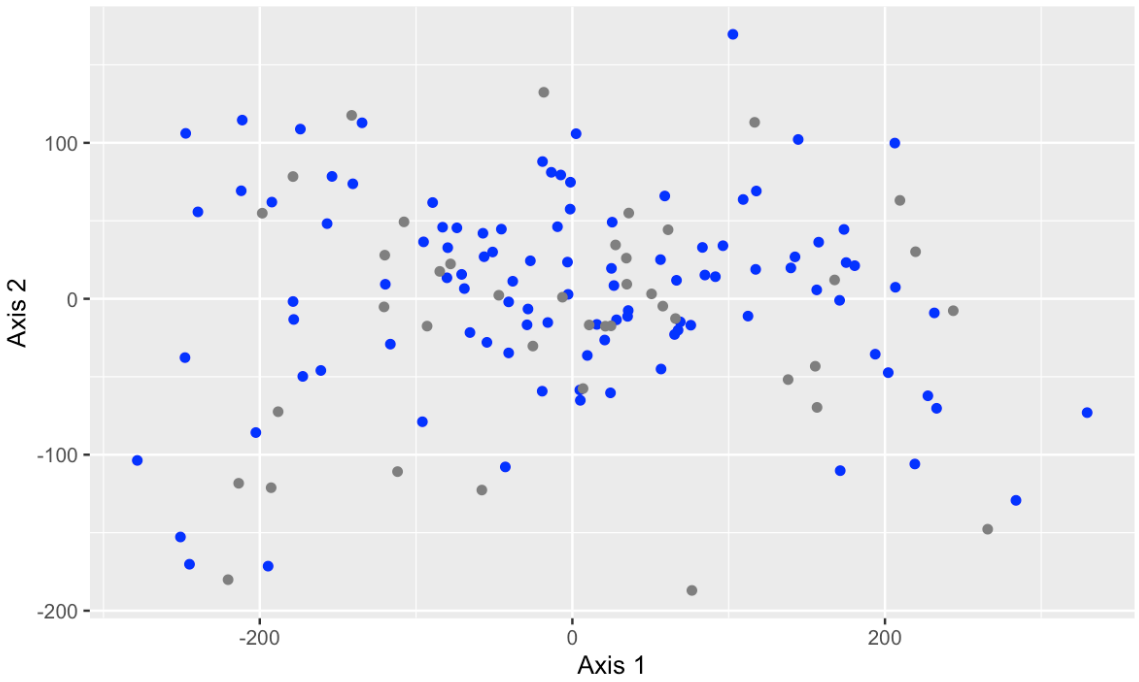


**Figure S1. MDS plots of covariate correction for array Version number.** Version 1 samples are shown in blue, and Version 2 samples in green. All CpGs in the Methylation Value matrix were used in the analysis. The cmdscale function in the stats package in R was used to generate the coordinates for each sample, and ggplot2 was used to create the plots. A. Top plot shows the uncorrected plot. B. Covariate correction was performed using the removeBatchEffect function in limma.
