# Supplementary material for: Distinct Regulatory DNA Methylation Signatures Across Multiple Sclerosis, Neuromyelitis Optica, and Neurological Post-Acute Sequelae of COVID-19
Source: J Clin Med. 2026 Jun 25;15(13):4968. doi: 10.3390/jcm15134968 (PMC13362688; doi:10.3390/jcm15134968)
Supplement: Supplementary file 1 [file jcm-15-04968-s001.zip › jcm-4336141-supplementary material/Supplemental Figures/Figure S2.pdf]

Figure S2

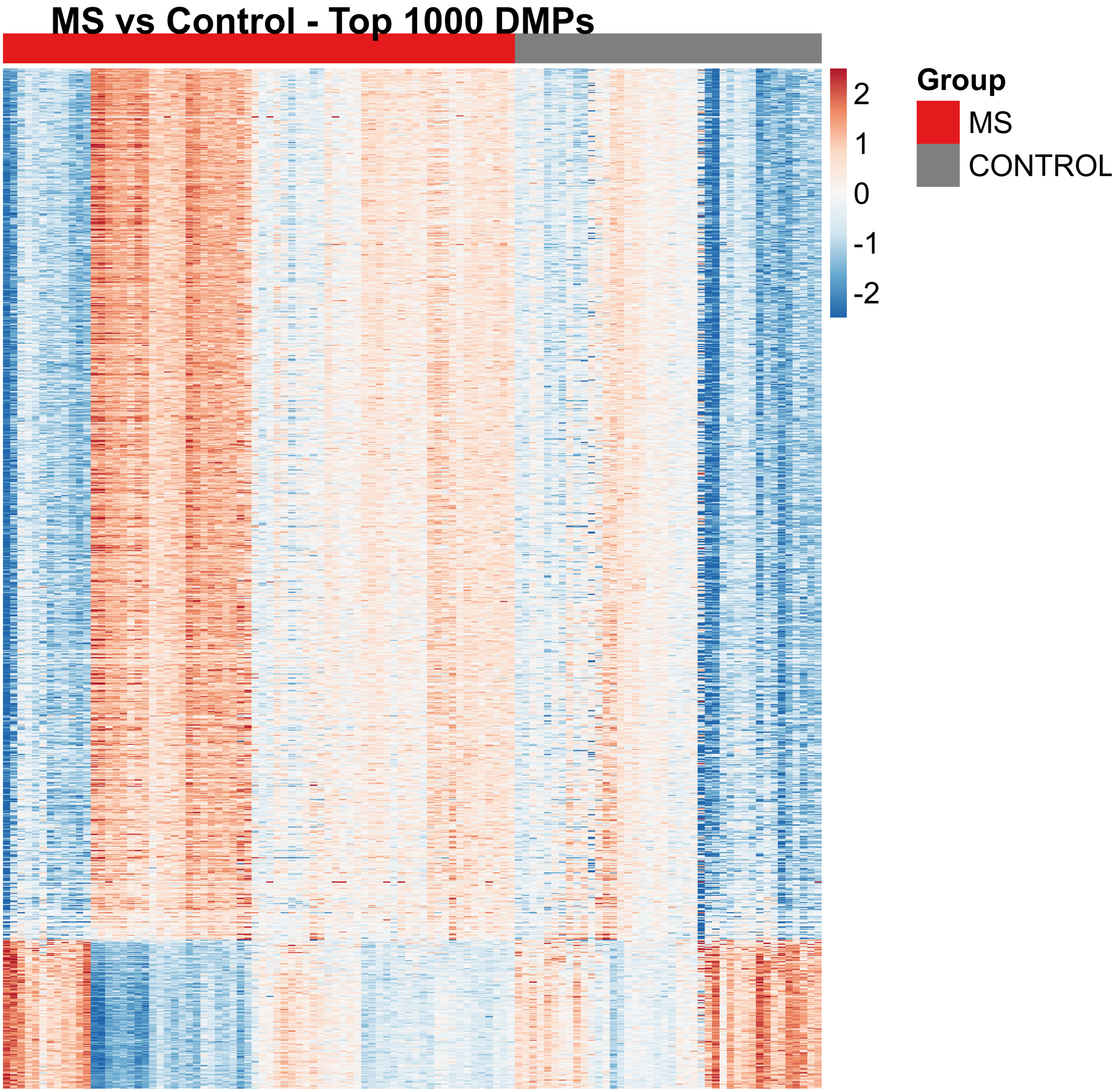

Figure S2. Heatmap of the top 1,000 MS-versus-control differentially methylated probes (DMPs). Heatmap of the 1,000 most strongly disease-associated CpGs identified in the MS-versus-control limma EWAS, ranked by adjusted p-value (rank_by_adj_p). Rows are individual CpG probes; columns are individual donors. Cell color encodes the row-wise z-score of the normalized β value (red, hyper-methylated relative to the row mean; blue, hypo-methylated relative to the row mean; z-scores capped at ±2.5 for color stability). CpGs are sorted vertically so that probes with the largest mean MS-minus-control β difference appear at the top (case-hypermethylated) and those with the most negative difference at the bottom (case-hypomethylated). Donors are grouped by phenotype (MS, n = 70, green annotation bar; CONTROL, n = 42, grey annotation bar) and clustered within each group using Ward.D2 hierarchical clustering on the z-scored matrix; sample identifiers and dendrograms are not displayed. The β-value matrix used here was filtered, funnorm-normalized, and adjusted in the limma model for race, EPIC array version, age, sex, disease-modifying treatment class, and EpiDISH-derived blood cell composition.
