# Supplementary material for: Distinct Regulatory DNA Methylation Signatures Across Multiple Sclerosis, Neuromyelitis Optica, and Neurological Post-Acute Sequelae of COVID-19
Source: J Clin Med. 2026 Jun 25;15(13):4968. doi: 10.3390/jcm15134968 (PMC13362688; doi:10.3390/jcm15134968)
Supplement: Supplementary file 1 [file jcm-15-04968-s001.zip › jcm-4336141-supplementary material/Supplemental Figures/Figure S3.pdf]

Figure S3

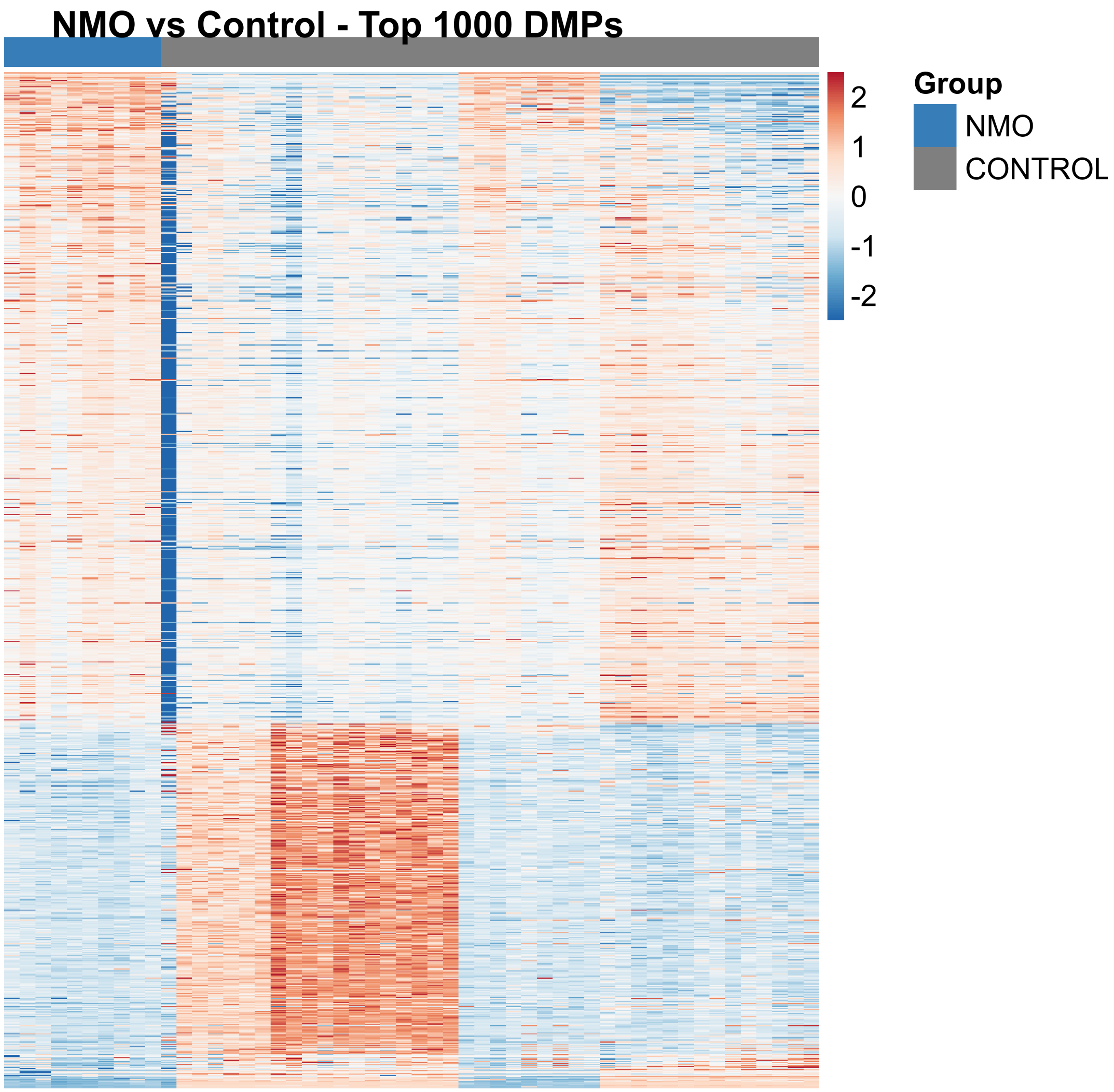

Figure S3. Heatmap of the top 1,000 NMO-versus-control differentially methylated probes (DMPs). Heatmap of the 1,000 most strongly disease-associated CpGs identified in the NMO-versus-control limma EWAS, ranked by adjusted p-value. Plot conventions are identical to Figure S2: rows are CpGs ordered by mean case-minus-control β difference (case-hypermethylated at top, case-hypomethylated at bottom); columns are donors grouped by phenotype (NMO, n = 10, blue annotation bar; CONTROL, n = 42, grey annotation bar), clustered within each group by Ward.D2 on the z-scored matrix. Cell color encodes the row-wise z-score of the normalized β value, capped at ±2.5. The same limma covariate adjustments were applied as for Figure S2 (race, EPIC array version, age, sex, disease-modifying treatment class, and EpiDISH-derived blood cell composition).
