# Supplementary figures and images for: Distinct Regulatory DNA Methylation Signatures Across Multiple Sclerosis, Neuromyelitis Optica, and Neurological Post-Acute Sequelae of COVID-19
Source: J Clin Med. 2026 Jun 25;15(13):4968. doi: 10.3390/jcm15134968 (PMC13362688; doi:10.3390/jcm15134968)

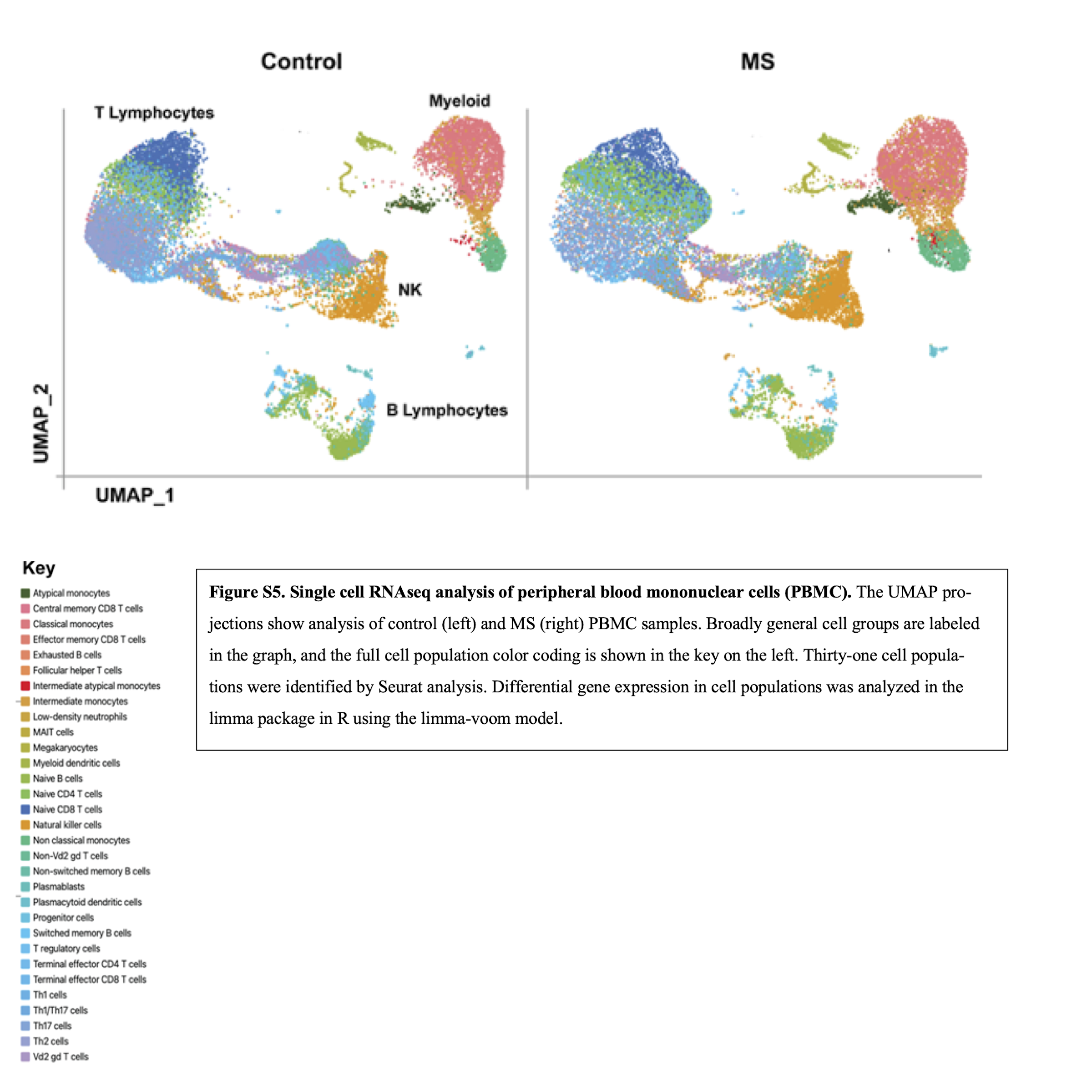

Supplement: Supplementary file 1 [file jcm-15-04968-s001.zip › jcm-4336141-supplementary material/Supplemental Figures/Figure S5.tiff]
